# Integrating open education practices with data analysis of open science in an undergraduate course

**DOI:** 10.1002/ece3.70129

**Published:** 2024-08-13

**Authors:** Marja H. Bakermans

**Affiliations:** ^1^ Department of Integrative and Global Studies Worcester Polytechnic Institute Worcester Massachusetts USA; ^2^ Department of Biology and Biotechnology Worcester Polytechnic Institute Worcester Massachusetts USA

**Keywords:** data repository, open access, primary literature, SALG, variable grading

## Abstract

The open science movement produces vast quantities of openly published data connected to journal articles, creating an enormous resource for educators to engage students in current topics and analyses. However, educators face challenges using these materials to meet course objectives. I present a case study using open science (published articles and corresponding datasets) and open educational practices in a capstone course. While engaging in current topics of conservation, students trace connections in the research process, learn statistical analyses, and recreate analyses using the programming language R. I assessed the presence of best practices in open articles and datasets, examined student selection in the open grading policy, surveyed students on their perceived learning gains, and conducted a thematic analysis on student reflections. First, articles and datasets met just over half of the assessed fairness practices, which increased with the publication date. There was a marginal difference in how assessment categories were weighted by students, with reflections highlighting appreciation for student agency. In course content, students reported the greatest learning gains in describing variables, while collaborative activities (e.g., interacting with peers and instructor) were the most effective support. The most effective tasks to facilitate these learning gains included coding exercises and team‐led assignments. Autocoding of student reflections identified 16 themes, and positive sentiments were written nearly 4x more often than negative sentiments. Students positively reflected on their growth in statistical analyses, and negative sentiments focused on how limited prior experience with statistics and coding made them feel nervous. As a group, we encountered several challenges and opportunities in using open science materials. I present key recommendations, based on student experiences, for scientists to consider when publishing open data to provide additional educational benefits to the open science community.

## Graphical abstract

This manuscript presents a case study using open science (published articles and corresponding datasets) and open educational practices in an undergraduate capstone course. In this course, students engage in current topics of conservation, trace connections in the research process, learn statistical analyses, and recreate analyses using the programming language R. I assessed the presence of best practices in open articles and datasets, examined student selection in the open grading policy, surveyed students on their perceived learning gains, and conducted a thematic analysis on student reflections.

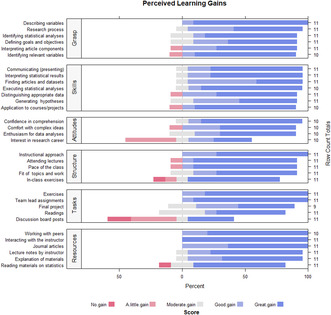

## 1 INTRODUCTION

The U.S. White House Office of Science and Technology Policy (OSTP) declared 2023 the Year of Open Science for all federal agencies (The White House, 2023). What does this mean, and what are its implications? Open science aims to make scientific research accessible to society by sharing all aspects of the scientific workflow – from discovery to analyses to outputs (Farrow et al., 2020; Tennant & Breznau, 2020). Open science has been described as embracing characteristics like transparency, accountability, rigor, equality, accessibility, reproducibility, reusability, and inclusivity (Alberts et al., 2015; Pownall et al., 2021; Tennant & Breznau, 2020). The open science movement has been successful because of the broad array of benefits to researchers and society, like the reproducibility of science (McKiernan et al., 2016; Tennant et al., 2016), preserving data for potential future use (Beck et al., 2020; Tennant et al., 2016), boosts citations (McKiernan et al., 2016; Tennant et al., 2016; Toelch & Ostwald, 2018), and greater media coverage, which can lead to more collaborations, funding, and career advancement (McKiernan et al., 2016; Toelch & Ostwald, 2018; Vicente‐Saez & Martinez‐Fuentes, 2018). Furthermore, open science provides broader access, especially for under‐resourced individuals and organizations (Hampton et al., 2015; Tennant et al., 2016; Vicente‐Saez & Martinez‐Fuentes, 2018) and can give access or voice to those who are often excluded in publishing (Arza & Fressoli, 2018; Grahe et al., 2020). Yet, open science comes with its potential limitations, challenges, pitfalls, and risks (Bahlai et al., 2019; Darda et al., 2023). In short, open science does not necessarily mean it is equally accessible or achievable, and differential systemic barriers exist based on career stage or position, finances, country, and cultural contexts (Bahlai et al., 2019; Darda et al., 2023; Martínez & Poveda, 2018; Ross‐Hellauer, 2022). Minoritized scientists (e.g., women, early career, BIPOC (Black, Indigenous, and People of Color), LGBTQ+, Global South) are most likely to experience negative repercussions of open science (Cech, 2022; Darda et al., 2023; Serwadda et al., 2018; Smith, 2001).

Fortunately, many sources have presented a range of guiding principles or best practices when it comes to the open science culture (Alberts et al., 2015; Darda et al., 2023) and practices (Barbosa et al., 2020; Kathawalla et al., 2021; Meyer, 2018; Wilkinson et al., 2016). To ensure data are usable and reproducible, it is recommended to document as much as possible in the process (Hampton et al., 2015) and use clean code and style guides to provide a consistent format or language (Barbosa et al., 2020; Filazzola & Lortie, 2022; Hampton et al., 2015; Jenkins et al., 2023). Wilkinson et al. (2016) lay out the FAIR data principles that consider the findability, accessibility, interoperability, and reusability of data to support the reuse of scholarly data. In sharing the research process, it is recommended that the authors consider the needs and skills of the audience so that the data and code are accessible to them or a broader audience (Purgar et al., 2022). Ethical considerations are recommended in implementing open science (Jenkins et al., 2023; Meyer, 2018). For example, blinding the research in data collection or analyses can improve the robustness of a study and reduce wasted (i.e., non‐reusable) data (MacCoun & Perlmutter, 2015; Purgar et al., 2022), and incentives and evaluation around publishing (Alberts et al., 2015; Barbosa et al., 2020; McKiernan et al., 2016; Purgar et al., 2022) need adjustments to ensure that a range of published products and sources are valued. Also, several sources offer guidance to support early career and historically minoritized groups in navigating and contextualizing a continuum of open science practices (Kathawalla et al., 2021; McKiernan et al., 2016; Pownall et al., 2021; Serwadda et al., 2018).

Open science can be placed in a larger open movement that also includes open education. Open education encompasses any pedagogy informed by the open movement or open access and focuses on sharing the effort and resources of instructors and learners (DeRosa & Robinson, 2017; Katz & Van Allen, 2022; Lambert, 2018; Witt, 2020). Open education and open science can share similar social justice aims of redistributing, recognizing, representing, and restructuring scientific knowledge and experience (Bali et al., 2020; Clinton‐Lisell et al., 2023; Derosa & Jhangiani, 2017; Lambert, 2018). Openly accessible and freely adaptable materials, like open textbooks, courses, and educational resources (OERs), enable faculty to customize and curate material aligned to course learning objectives while including current events and being culturally relevant and representative of a diverse set of expertise. These elements aim to make scientific knowledge accessible to society and may challenge established scientific pedagogy (Farrow et al., 2020). The use of these materials has been shown to increase student scholarly identity, agency, and sense of belonging in STEM fields, especially for students from marginalized populations (Clinton‐Lisell et al., 2023; Hays & Mallon, 2021; Nusbaum, 2020). OERs, representing any aspect of the teaching and learning curriculum (e.g., lectures, activities, sources, assignments, etc.), are increasingly used in curriculum and on STEM‐focused platforms (e.g., CourseSource).

Furthermore, open educational practices describe any method arising from an open pedagogical approach (Bali et al., 2020; Witt, 2020). By this definition, open education practices draw from connectivism (Downes, 2022; Siemens, 2005) and learner‐centered theories (Mccombs, 2001) and can elevate social justice elements of open science (Bali et al., 2020; Clinton‐Lisell et al., 2023; Katz & Van Allen, 2022). Open education practices include “practices that are based on a competency‐focused, constructivist paradigm of learning and promote a creative and collaborative engagement of learners with digital content, tools and services in the learning process” (Schaffert & Geser, 2008, p. 3). In short, open education practices are concerned with learner agency and outcomes in the learning process (Bali et al., 2020; Witt, 2020).

In the context of a capstone course with upper‐level students who are considering their career goals, using primary literature can engage students in real‐world topics and applications while fostering scientific practices (Rauschert et al., 2011). The exponential rise in openly licensed primary literature and associated datasets (Coudert, 2020; Piwowar et al., 2018; Pourret & Ibarra, 2023; Tennant et al., 2016) can potentially represent an enormous OER. However, teaching with primary literature can be time‐consuming, and students often struggle with understanding the big picture, data interpretation, and scientific jargon and techniques (Chatzikyriakidou & McCartney, 2022; Howard et al., 2021; Nelms & Segura‐Totten, 2019). Despite advancements, researchers and educators are only beginning to embrace the synergies of open science and open education practices (Burgos, 2020; Grahe et al., 2020; Kessler et al., 2021). For example, there is a need for training early career researchers and integrating open science practices into graduate curricula, while emphasizing reducing and removing systemic barriers for those most impacted (Barbosa et al., 2020; McKiernan et al., 2016). To support this transformative system, there also must be research into ways to improve scholarly development, use, and communication of resources (Barbosa et al., 2020; Tennant et al., 2016). Lastly, more open science education is required, especially with a social justice lens (Grahe et al., 2020; Toelch & Ostwald, 2018).

### 1.1 Goals of this study

Open science is still primarily research‐focused and centered on data, code, notes, publications, and other outputs (Farrow et al., 2020) and lags in integration with open science education and pedagogy (Grahe et al., 2020; Kessler et al., 2021). This study examines the combination of open science and open educational practices in training and educating STEM undergraduate students. Using a capstone course as a case study, I aim to explore the challenges and opportunities of using open science in an undergraduate course. I first examined the extent to which articles and datasets used for this course followed best practices in open science. Next, I examined how students responded to open education practices, like determining the weights of course assessments toward their final grade. Third, I surveyed students on their perceived learning gains related to course objectives, structures, and activities. Lastly, in reflections on their learning, I investigated autocoded themes and their sentiments while considering connections to open science and course grading.

## 2 CASE STUDY

*Analyses in Wildlife Ecology and Conservation* is a capstone course where students use primary literature to learn and apply data analysis techniques in wildlife ecology and conservation. At this institution, a capstone is an advanced‐level course designed to integrate concepts from across the curriculum and practice skills in analyzing, evaluating, and communicating scientific information. This was a fee‐free course, where students did not purchase additional materials. In this capstone, students examined openly published datasets analyzed in recent journal articles. Articles and their datasets were selected to include a range of topics and authors from across the globe (e.g., spanning five continents) by first searching open data repositories (e.g., Dryad) by statistical tests. After completing this course, students should be able to (1) summarize current issues in wildlife ecology and conservation, (2) illustrate the development of and connections in the research process (goals – research questions – hypotheses – variables – analyses – results), (3) breakdown components of biological data analyses, (4) recreate and apply the research process and data analyses in R using open data and articles, (5) engage in self‐directed learning, and (6) demonstrate knowledge and skills developed in the area of expertise of a project. Designed as an advanced course for biology or related majors, the course population limit is set to 15 students and is targeted for fourth‐year students. This course facilitated student‐controlled learning environments by integrating collaboration and mentoring structures, allowing students to choose materials for lead and final project assignments, and fostering agency in grading policies.

### 2.1 Course structure

This capstone course was taught twice weekly, with each class lasting 1 h and 50 min over 14 sessions (Appendix A). Sessions 1 and 2 were designed as background and introduction to the course, coding in R, and open datasets. From Sessions 3 through 12, that week's days 1 and 2 were paired by the topic, activities, and assignments. On the first day, I introduced and discussed statistical analyses, with examples of research questions, codes, results, and interpretations of those results. For this class, I used the dataset from the previous week (so students were familiar with it), and I shared my lecture slides with all students. Students prepared for this class by reading information on statistical analyses before class. The remainder of day 1 was spent by students working on the exercise, due on day 2's end, while I assisted them. Typically, students worked through the initial components of the coding exercise. In the second class of the week, one team led a discussion around the article and analysis of the week and provided additional information (on a new article), with the rest of the class as participants. Students were expected to be familiar with the assigned article of the week before they came to the week's first class.

### 2.2 Coursework

#### 2.2.1 Coding exercises

Students were assigned six exercises across the 7‐week term. These consisted of three parts: (a) working through the background context of the article's study, (b) exploring the corresponding dataset, recreating analyses in R coding, and reporting results, and (c) summarizing student results and explaining their implications. In part a, students were required to identify the study's goal, research questions, and hypotheses while identifying and describing the variables analyzed. Part b focused on learning and executing different weekly statistical tests and methods. Students were given class time to ask questions (from me or peers) but were also expected to complete exercises outside of class.

#### 2.2.2 Lead exercise

For the lead exercise, students worked in small teams to present the article, dataset, and coding exercise of the week selected by the instructor and explained another article that they selected and its related analysis. Students were instructed to find the same statistical tests in this new article by searching data repositories. Presentations by each team were expected to last between 40 min to 1 h, and students submitted the presentation slides.

#### 2.2.3 Open annotations

Students engaged with sources (i.e., journal articles and textbook chapters on statistical analyses) and their peers using social annotation software (Perusall®). Students were expected to read and clarify text passages, share perspectives, interpretations, and additional related information, ask and respond to questions, and connect concepts across topics. I (and other colleagues) have used this social annotation tool in other courses at this STEM institution, and we have found that it can promote more equitable interactions across students, especially for historically minoritized students, by deepening knowledge and engagement with the source and their peers and redistributing epistemic authority (Bakermans et al., 2022). Students accessed the social annotation platform through our campus LMS (Learning Management System).

#### 2.2.4 Final project

For the final project, students worked individually and used one new article and dataset on the course topic. Students were advised to search data repositories using statistical keywords to find articles and datasets based on the target analyses. Students had to work through similar components (parts a, b, and c) as described in the coding exercises. However, they did this for one statistical test they struggled with earlier and a new analysis not taught during the course. The objectives of the final project were for each student to review past work, apply their knowledge to a new situation, and explore a more complex statistical analysis.

#### 2.2.5 Discussion board and statement of learning

Students used a discussion board on the LMS to post questions, answers, and comments to fellow students while working through the term's assignments. The discussion board was designed to allow students to troubleshoot issues during coding exercises or projects. Each student was expected to write at least two original posts and comment on at least three posts written by others. Also, at the end of the course, students were asked to reflect on their learning about the topic, process, and themselves throughout the course in a “Statement of Learning” assignment. In assignment instructions, students were prompted to consider what areas of growth or improvements they recognized as a result of this course. I provided the flexible guidelines of a one‐ to two‐page written reflection.

#### 2.2.6 Attendance and participation

Students were expected to attend and participate positively and professionally as expected of a college student and contribute to all activities in a purposeful manner that ensures that the course objectives are reached.

## 3 METHODS

### 3.1 Article and dataset fairness

To assess the utility of open articles and their datasets as an educational tool in an undergraduate academic setting, I measured the congruence of each pair (article + dataset) to a set of best practices and guiding principles. I assessed ten guiding principles and best practices (Table 1), where each category was scored “1” or “0” based on whether it met that criteria, with a total possible score of ten. Eight categories were selected from the most relevant FAIR Principles, which aim to improve the usability of scholarly data (Wilkinson et al., 2016). These principles relate to findable, accessible, interoperable, and reusable data. Two additional categories assessed best practices in including and clarifying coding scripts (Filazzola & Lortie, 2022; Jenkins et al., 2023). Fairness principles and best practices were applied to all articles and datasets which were selected by the instructor and students.

**TABLE 1:** Datasets from articles were assessed for best practices based on selected guiding principles of FAIR (#1–8 above; Wilkinson et al., 2016) and best practices from Jenkins et al. (2023; #9) and Filazzola & Lortie (2022; #10).

Principle or practice	Code	Descriptor
1. Findable	F1	Data are assigned a unique and persistent doi
2. Findable	F2	Metadata include an identifier of data
3. Findable	F3	Data are registered in a searchable database
4. Accessible	A1	Data are retrievable by their identifier
5. Accessible	A2	Metadata are accessible and retrievable
6. Interoperable	I1	Data use an accessible and shared language
7. Interoperable	I2	Data include qualified references to other (meta)data
8. Reusable	R1	(Meta)data are described with accurate and relevant attributes
9. Coding	C1	Coding scripts, etc. used for analyses are included
10. Coding	C2	Coding scripts provide an adequate explanation of the steps

In addition, I assessed if the data needed for the articles' analysis was included in the dataset (i.e., coherence of data and analyses). For this step, I only examined the articles and datasets used in the course exercises (*n* = 6) and the statistical tests highlighted in the course that week, not all the statistical tests run by the authors. For example, if students were learning and expected to recreate a *t*‐test in that week's coding exercise, I selected a journal article that used a *t*‐test in their statistical data analyses. I analyzed these data using descriptive statistics.

### 3.2 Open grading policies

Based on universal design for learning and open pedagogical approaches (Kaw, 2020), students were allowed to specify the percentage weight for each assessment category that contributed to their final grade, with the constraint that no single category can be <10% and no single category can be >40%. Assessment categories of the course included (1) six coding exercises (Exercises), (2) one lead exercise (Lead Exercise), (3) 14 annotation assignments of readings (Annotations), (4) one final project (Final Project), (5) five discussion board posts and a statement of learning reflection (Discussion), and (6) attendance and participation (Participation).

Students were asked to indicate the grading weights of their choice by the course's midpoint. Alternatively, a student could choose the grading distribution that I set for the class before the course started, which was as follows: Exercises: 30%, Lead Exercise: 25%, Open annotations of sources: 15%, Final Project: 10%, Participation & Attendance: 10%, and Reflection + Discussion Board posts: 10%. I tested if students chose equal weights (dependent variable) across evaluation assessment categories (independent variable) using an analysis of variance (ANOVA) and examined pairwise differences with Tukey's HSD. In addition, to visualize the difference between student and instructor decisions, I plotted student weights next to the instructor grading scheme.

### 3.3 Assessment of perceived learning gains

I used a student assessment of learning gains (SALG) survey to measure students' perceptions of learning gains related to course objectives (Seymour et al., 2000). This instrument is registered as #113953 at https://www.salgsite.net. This Likert‐scale survey provided five response categories ranging from “no gains” to “great gains” in learning and the option of open responses in each category. Categories included in the survey connected to the following: understanding of course content, increases in your skills, course impact on attitudes, integration of their learning, the course, course activities, assignments and graded activities, course resources, the information they were given, and support for students as individual learners. Likert‐scale data were visualized using the Likert function (HH package; Heiberger & Robbins, 2014). A summary report that converted Likert responses to numbers and calculated descriptive statistics (Lovelace & Brickman, 2013) was produced from the SALG instrument website.

### 3.4 Student reflections

In student reflections, I examined the frequency of the 100 most frequent words, with stop words excluded and a minimum length of four (letters), both “with synonyms” and “with generalizations.” Including synonyms allows grouping terms with the same stem and close meanings (e.g., help, helpful, assist, support). Allowing generalizations broadens the scope to include words with a more general meaning (e.g., help, helpful, assist, improve, advance). Next, I used thematic analysis, “a method for identifying, analyzing, and reporting patterns (themes) within data” (Braun & Clarke, 2006), on student reflections. Due to this paper's explorative nature, I used autocoding to identify students' broad themes and sentiments in their reflections. Autocoding examines the sentiment of each word and scores it as positive, neutral, mixed, or negative. In this process, I compared how students felt about each theme, focusing on positive (i.e., satisfaction) and negative (i.e., dissatisfaction) sentiments. The relationship of how sentiment was coded to themes was visualized in a tree map, where the size of a block is relative to the number of references for that code. All reflection processing and analyses were performed in NVivo 14 (Windows).

All data were collected with institutional IRB approval (IRB‐24–0314). All statistical analyses were performed in R (ver. 4.3.1; R Core Team, 2023). Residuals from all models were examined for normality and heteroskedasticity by visually examining histograms and normal probability plots. Data from this study can be found at https://doi.org/10.5061/dryad.37pvmcvst.

## 4 RESULTS

### 4.1 Student population

Thirteen students enrolled and completed the capstone course, with 12 as Biology and Biotechnology majors and the other as a Bioinformatics and Computational Biology major. In addition, several students were pursuing a second major (Environmental and Sustainability Studies, Professional Writing) or a minor degree (History, Spanish, Biochemistry). Ten were fourth‐year students, and the remaining three were third‐year students when completing the course.

### 4.2 Article and dataset fairness

This course used 22 published peer‐reviewed journal articles and their corresponding datasets (*n* = 6 for class exercises, *n* = 4 for student‐led presentations, and *n* = 12 for individual student final projects; Appendix B). All datasets were openly published with Creative Commons licenses, most of them (22/23) stored in the Dryad data publishing platform (https://datadryad.org).

**FIGURE 1:**
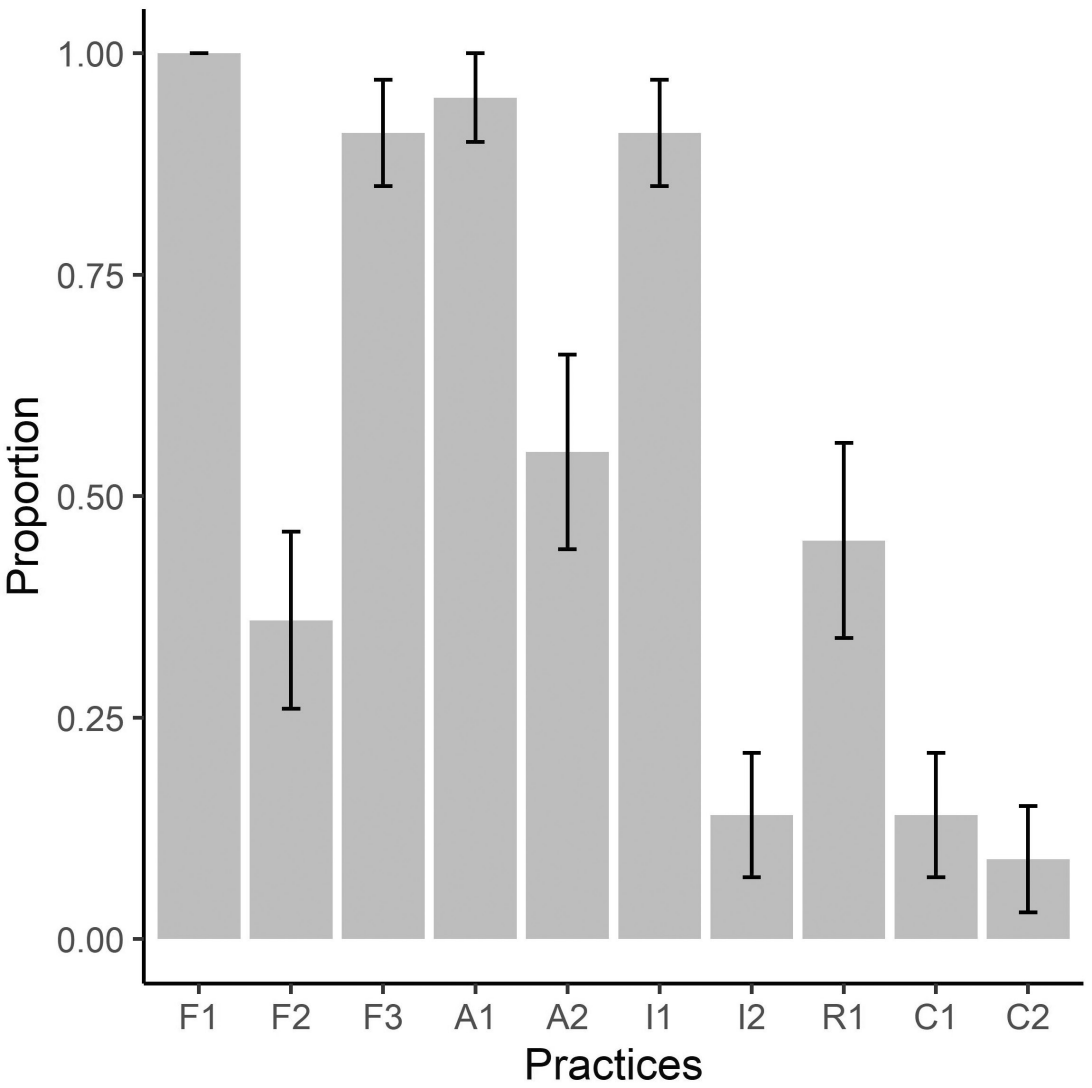
Bar graph representing adherence to best practices in open data sharing across the 22 journal articles and their corresponding datasets used in the course. See Table 1 for abbreviations and explanations of each category.

On average, datasets adhered to half (i.e., 5.5 out of 10, 0.4 SE) of the fairness practices outlined in this paper (Figure 1). All datasets (22/22) followed the findable guideline of being assigned a unique and persistent identifier. However, few datasets included interoperable information including qualified references to other (meta)data (*n* = 3), coding script used in analyses (*n* = 3), and explanation of coding steps in the script (*n* = 2). Three datasets (i.e., 50%) used in conjunction with the course exercises did not follow the coherence of dataset and article analyses – that is, the datasets did not include data that the authors used in their published analyses. The publication dates of datasets used in this course ranged from 2015 to 2023, with 11 published in 2022 and 2023.

### 4.3 Grading policy

**FIGURE 2:**
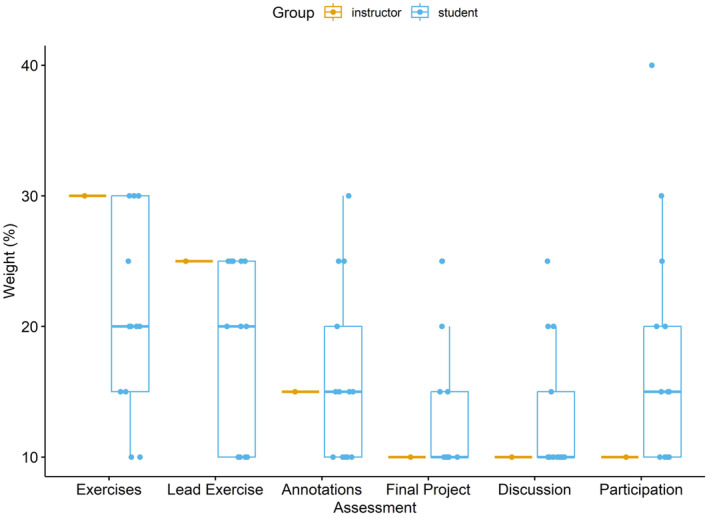
Boxplots and data points of student choice (blue) of percentage weights of assessment categories compared to instructor (yellow) weights.

There was no evidence to suggest that the variance across assessment groups differed significantly, but there was a slight deviation from normality. However, ANOVA is not sensitive to slight deviations from this assumption (McDonald, 2014), so I retained this parametric analysis. The percentage weight for assessment categories ranged from 13.1% (1.4 SE) for Discussion to 21.2% (2.1 SE) for Exercises (Figure 2). There was a marginal difference in how the assessment categories were weighted by students (*F*_5,72_ = 2.33, *p* = .051) with a significant difference between the Discussion and Exercises assessment categories (adjusted *p* = .050). Compared to the grading scheme selected by the instructor, students gave lower weights to work that involved initial coding and statistical tests (i.e., Exercises and Lead Exercise) and higher weights to final projects, participation, and discussion assessments (Figure 2).

### 4.4 Student learning gains

**FIGURE 3:**
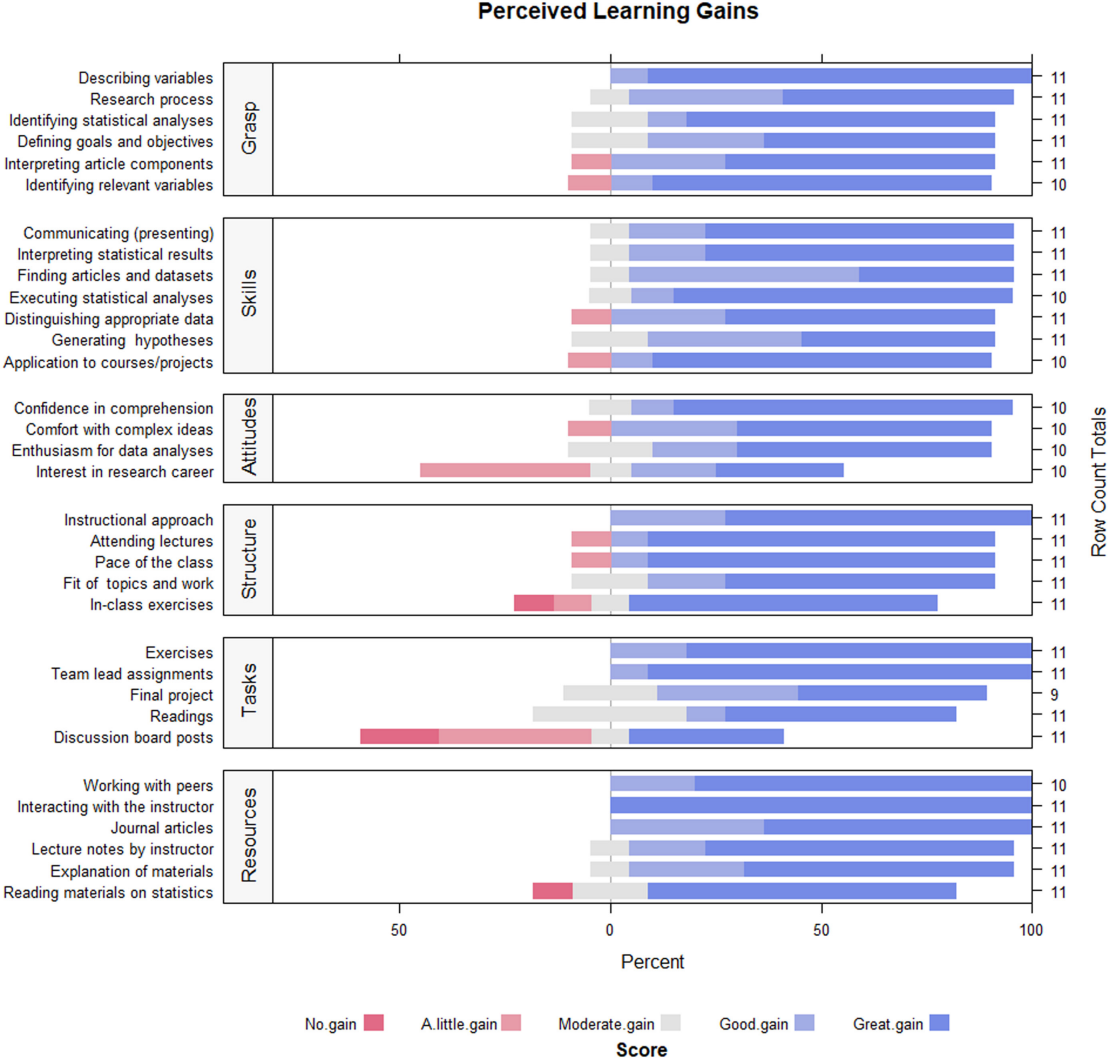
Self‐reported perceived learning gains from students upon completion of the course. Students assessed their learning gains as they related to (1) understanding of course content (Grasp), (2) increase in their skills (Skills), (3) impact of their attitudes (Attitudes), (4) course structure (Structure), and (5) assignments (Tasks), and support and resources offered to them (Resources). See instrument #113953 at https://www.salgsite.net for survey questions.

Eleven of 13 (i.e., 85%) students completed the SALG survey after the course. Students generally reported good to great gains across course elements (Figure 3). In particular, students found support from the instructor (5.0 ± 0.0) and their peers (4.8 ± 0.42) to be especially helpful and indicated that the instructional approach provided great help in their learning (4.7 ± 0.5). Regarding the most useful course activities, students indicated that team‐led assignments (4.9 ± 0.3) and exercises (4.8 ± 0.4) provided the most help in their learning. In contrast, the discussion board posts provided the least help (3.0 ± 1.7) in their course learning. Furthermore, students reported that they gained the most understanding in describing types of variables (4.9 ± 0.3) and gained the most gains in the skills of executing statistical analyses in R (4.7 ± 0.7). Student attitudes in their confidence in comprehension of materials had a large gain (4.7 ± 0.7). Although enthusiasm for data analyses saw large gains (4.4 ± 0.8), there were moderate gains in students' interests in a data analysis career (3.4 ± 1.4).

### 4.5 Student reflections

#### 4.5.1 Autocoded themes and sentiments

The most frequently used words in student reflections when considering synonyms were connecting activities and concepts (e.g., “coding” and “statistical”) to how they perceived those components' utility in the course (e.g., “helped” and “learned”; Table 2). When considering generalizations, the most commonly mentioned words identified a few additional words that illustrate the action verbs of students in the course. In particular, the words “actively” and “communicating” demonstrate how students found the work “educational” (Table 2).

**TABLE 2:** Summary of the top seven words with synonyms (left) and generalizations (right) generated from autocoding student reflections.

Word	With synonyms		Word	With generalization	
	Count	Weighted percentage (%)		Count	Weighted percentage (%)
Course	194	3.84	Actively	529	1.60
Learned	181	3.54	Statistical	60	1.25
Statistical	56	1.54	Educational	274	1.14
Coding	53	1.46	Learned	229	1.10
Tests	55	1.40	Tests	92	1.07
Helped	48	1.22	Work	309	0.93
Class	85	1.21	Communicating	404	0.91

Autocoding identified the following 16 themes: statistical (*n* = 35 references), tests (*n* = 24), research (*n* = 22), course (*n* = 16), coding (*n* = 16), data (*n* = 13), biology (*n* = 12), paper (*n* = 12), analysis (*n* = 11), learning (*n* = 9), class (*n* = 9), reading (*n* = 9), experience (*n* = 8), topics (*n* = 8), ecology (*n* = 8), and article (*n* = 7). These themes primarily relate to the goals and objectives of the course (e.g., statistical research and analyses), class activities (e.g., coding data and reading articles), class resources (e.g., journal articles or papers), and course topics (e.g., ecology, biology). The codebook created in the autocoding process revealed that each theme included between 5 (e.g., “articles” theme) and 16 (e.g., “coding” theme) subthemes. For example, the theme “analysis” included the words or phrases “actual analysis,” “data analysis,” “data analysis articles,” “data analysis techniques,” “different analysis,” “literary analysis,” “statistical analysis,” and “statistical analysis results.”

**FIGURE 4:**
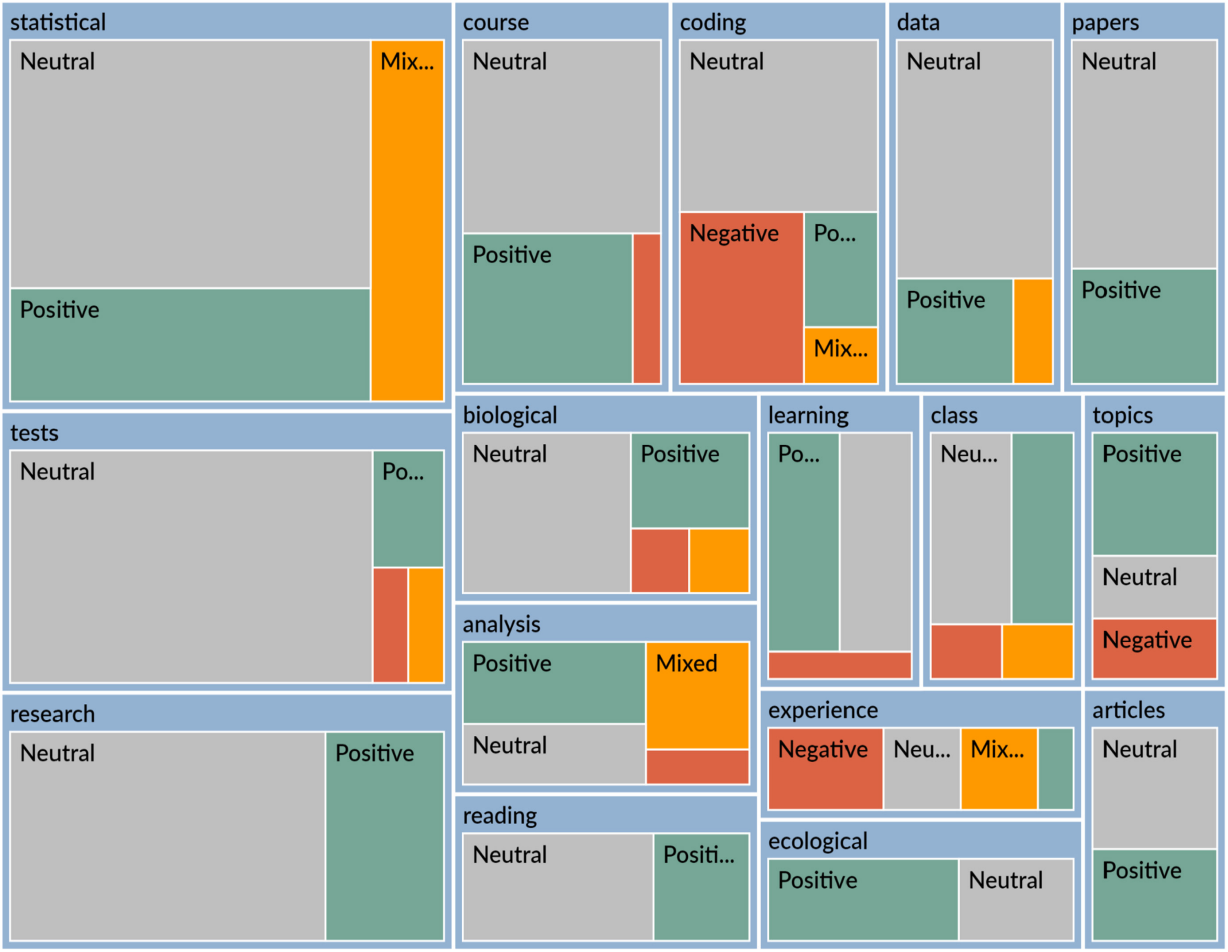
A tree map that visualizes how sentiment is coded to themes, where the size of a block is relative to the number of references for that code. Sentiment is recorded as positive (green), neutral (gray), mixed (orange), and negative (red). This tree map was created in NVivo 14.

The tree map (Figure 4) illustrates 438 sentiments coded to themes. Neutral sentiment was the most common (*n* = 344 items coded, 79%), followed by positive (*n* = 62, 14%), negative (*n* = 16, 4%), and mixed (*n* = 16, 4%). The theme with the most coded sentiments was “statistical” with 70 references.

Instances in reflections coded as neutral sentiment typically provided facts about the structures or processes of the course. An example sentence coded as neutral was “After we had collected sufficient data, our reports and writing shifted to include statistical analyses.” Another example was, “Many of the statistical tests which I have learned about were also new to me, such as the nonparametric Wilcoxon signed‐rank test for comparing one measurement and one nominal variable (sic).” The theme of “statistical” had the most mixed sentiments (*n* = 6). Instances coded with mixed sentiment had both positive and negative terms identified. Most often, this was when students voiced their initial apprehension of the course while providing an element of their growth due to the course. For example, one student wrote, “However, this course has greatly changed my perspective, especially during a period when I perceived statistical concepts as stressful and confusing.” Another mixed sentiment for “statistical” was included: “Literary analysis is often not a focal point in the statistical classes I have taken in the past, but proved to be very beneficial in my learning in this course.”

The themes that had the most negative sentiments were “coding” (*n* = 5), “experience” (*n* = 3), and “topics” (*n* = 2). Students discussed their negative sentiments (e.g., struggles and concerns) with “coding” in experiences before the course. For example, one student wrote, “I came into this class fairly scared considering I had essentially no coding experience whatsoever.” Similarly, students reflected on how their limited past “experience” with statistics and coding made them feel on the first day of class. This sentence exemplifies how students felt, “Prior to taking this course, I had very little experience with programming and coding, so I was a little nervous about taking a class that focused on coding.” Finally, autocoding of negative sentiments related to “topics” seemed to mischaracterize a negative word to indicate that was the student's feeling. For example, in the statement, “In particular, I felt like I learned a lot about current topics in wildlife ecology and conservation such as the human‐wildlife conflict,” the word “conflict” was miscoded as a negative sentiment.

The themes that had the greatest number of positive sentiments were “statistical” (*n* = 9), “research” (*n* = 6), and “course” (*n* = 6). Words or phrases that provided a positive sentiment included “happy to know,” “I feel confident,” “grateful,” “broadened my knowledge,” “reinforced my grasp,” “competent,” “enjoyed,” and “well rounded.” In general, students were reflecting upon the growth in connection to their ability with “statistical” analyses. For example, one student wrote, “By the end of the 7‐week term, I feel confident that I can now engage in conversation with peers, professors, and others on what statistical tests could be used in certain experiments and what the statistical analysis results indicate the testing that was done.” Students also reflected on how components of the course positively reinforced their abilities in the “research” process. An example quote includes, “The ability to apply practical knowledge to solve problems is mentioned frequently in the learning objectives of different courses, but I found that the transfer of knowledge and the application of new skills to research questions was solidified in this course.” Finally, the positive sentiments as related to the “course” theme were typically commenting on course structure and materials. For example, one student wrote, “I learned a lot in this course and found the course material very helpful.”

#### 4.5.2 Themes and sentiments of open science and education practices

Comments related to the grading policy were often connected to the “learning” theme. For example, one student wrote, “Sometimes, I think class grading at WPI doesn't represent individual learning styles well because not all students excel in the same way.” Another student commented, “Having the freedom of customizing my grading percentages allowed me to fully immerse myself in the class learning environment rather than hyper fixating on a grade everytime (sic) it was released into Canvas.” Two students explained the strategies behind their weight choices by writing, “I liked that I was able to weigh things in a way that made me less nervous about achieving a specific ending grade and instead allowed me to focus on learning to my best ability, which often included making mistakes that impacted my assignment grades” and “I made the R exercises a relatively low weight in my grading activity assignment because I am not strong in coding and I wanted to be able to try new things and take risks in my R code without having to worry that it would be detrimental to my cumulative score.” Other students commented on elements like a sense of safety and collaboration that promoted their learning. For example, “I've come to realize that my optimal learning environment is one where I feel safe, unjudged, and supported by a patient professor who takes the time to explain concepts thoroughly.” Another student commented, “As a student, I have found that I learn the best in spaces that promote collaborative learning.”

A mix of sentiments was connected to concepts of open science fairness and was most often situated in the themes of “data,” “papers,” and “articles.” For example, negative sentiments were expressed in the comment, “I found myself extremely frustrated at times, whether it be with selecting variables or when encountering persistent error messages that made no sense to me.” Another commented on their difficulty in reading journal articles with, “I found these readings to be too complicated and confusing.” Additional frustration was written, “While others did not state their purposes clearly and many did not label or include sufficient data from their research to recreate their results.” Another student expressed, “While I understand this is partly a question of intellectual property, I think labeling variables that were versus were not used in an analysis and of course defining what abbreviations of variables mean are important for readers to replicate and learn from the study.”

Several comments connected the effectiveness of open education practices with their ability to learn about open science. For example, a student wrote, “Sharing example code with peers and discussing the research papers in class was the best way I have been able to learn about new environmental issues and R code these past 7 weeks.” Another student commented, “Reading the paper individually combined with discussing it as a class and hearing a group presentation on it really helped me draw connections between the biological data from the Dryad datasets and conservation importance.” Finally, a few students reflected on how lessons from this course will influence their future application, such as “As someone who has been trying to learn about statistical tests and sift through many papers, I will remember these experiences and try to provide clarity and data availability when I publish papers in my career.” Another similar comment includes, “As a byproduct of this, I realized what information is important to include in papers that I may publish in the future so that the audience gets the best reading experience and understanding of the experiments I conducted.”

## 5 DISCUSSION

This case study demonstrates that open science and education practices can serve as effective tools in undergraduate education because they can address multiple aspects of social justice (e.g., cost, recognition, agency) while meeting course objectives in scientific practices. Educators use primary literature to teach critical thinking and reading, science process skills, cutting‐edge science and techniques, real‐world problems, and ethics (Burnett et al., 2014). Reading and interpreting scientific literature is necessary for scientists and requires repeated instruction in education (Lee et al., 2022). In short, the goals of this course were to use open literature and data to facilitate student learning in the research process, learn and execute relevant statistical analyses, and build self‐efficacy while engaging in current issues in wildlife ecology and conservation. Students reported the greatest learning gains in describing types of variables, connecting components of the research process, communicating in the discipline‐appropriate style, executing and interpreting statistical results, and finding articles and datasets. These gains are in some of the most commonly reported areas where students struggle with reading and using scientific literature. For example, students tend to struggle with understanding the big picture, data interpretation, and scientific jargon and techniques (Chatzikyriakidou & McCartney, 2022; Howard et al., 2021; Kovarik, 2016; Nelms & Segura‐Totten, 2019; Rauschert et al., 2011).

In recreating statistical analyses, students struggled with generating biologically meaningful hypotheses and analyses that were relevant and testable to a dataset, so additional class time was dedicated to discussing and practicing these skills. This likely occurred because they were focused on the coding and did not understand the nature of the data itself, perhaps due to a lack of metadata or as novices in the topics. Students were most familiar and successful with numerical data and associated tests (e.g., correlations and linear regressions). However, some students struggled to recreate tests that required nominal and ranked variables (e.g., Chi‐square test of independence). In addition, some students struggled with slightly more complicated analyses, like when using three variables (e.g., multiple regression) or multiple steps (e.g., ANOVA followed by a multiple comparisons test). However, not all datasets could initially accommodate the set of statistical tests that students were to recreate, so students would have to rework the dataset (e.g., categorize a numerical variable into a nominal variable). I could have reworked datasets beforehand, but I wanted students to experience downloading raw data from the repository. As a trade‐off, in class, I worked through examples of creating a biologically relevant group from a numerical variable and discussed the nature of data collection, storage, and reproducibility with students. Also, the time constraint of implementing this course in a 7‐week term caused the need for multiple analyses (typically three) to be clumped in each week. If running this course over a more extended time, I would suggest reducing the number of analyses to recreate each week, with no more than two new analyses. Furthermore, spending more time finding articles and datasets that fit each test would be helpful, but it is difficult and time‐consuming. Most journal articles have analyses that are far too complicated for the majority of undergraduate students, so searching for articles and datasets by statistical test (e.g., ANOVA) was the most efficient.

This study exemplifies that open science and open education can facilitate the use of multiple instructional strategies to meet learning objectives. Educators use multiple approaches to facilitate using and learning from primary literature, including avoiding primary literature early in their academic career (Wise, 2021), employing different strategies depending on career stage (Hubbard & Dunbar, 2017), using social annotations (Bakermans et al., 2022; Kararo & McCartney, 2019; Washburn et al., 2023), peer‐to‐peer teaching (Theobald et al., 2017), and implementing a low stakes environment, like a journal club (Kozeracki et al., 2006). Others use a data‐centric approach where they focus on interpreting data or figures (Hoskins et al., 2007; Hunter & Kovarik, 2022; Round & Campbell, 2013). In this capstone course, I included multiple strategies, like social annotations, peer‐to‐peer teaching, and a data‐centric approach. The most effective tasks students identified in facilitating their learning gains included coding exercises and team‐led assignments. Given the course focus on the research process and data analysis, combined with students' limited prior experience with coding and analysis, as reported in the reflection statements, it makes sense that students would value the coding exercises. Also, most negative sentiments expressed in reflections were connected to fears of coding, using R, and statistical analyses at the start of the course due to their lack of or bad prior experience. Also, the collaborative team‐led assignments required students to conduct peer‐to‐peer teaching.

Students found discussion boards to be the least effective, but they may have initially been uncomfortable asking their peers for help. This initial apprehension may be related to a common misconception that science is a solitary act (McComas, 2002) or the discomfort of asking their peers (Rogerson & Scott, 2010). Alternatively, students may have perceived discussion boards as a lower‐quality peer collaboration tool. For example, lengthy lag time may occur between when a student posts a question and when or if another student responds, which can reduce a student's ability to solve a problem (Washington II et al., 2011). Indeed, students reported that the most effective classroom approaches that supported their learning were collaborative techniques, like working with peers and the instructor. This result is significant because it has been shown that students' fears and anxieties decrease in a classroom when provided multiple modes of learning and when given opportunities to learn from each other (Downing et al., 2020). Students also referred to collaboration positively in their reflections when it was connected to their learning in the course. Undergraduate students can face similar fears and barriers in open science as marginalized populations, and Truan and Dressel (2022) noted that students feared uncontrollable online visibility, vulnerabilities around the transformation of student to researcher, and harm to future careers. However, participating in open science retroactively gave students a sense of belonging in the scientific community.

Open education practices that are student‐centric can ease students into understanding and navigating the world of being a scientist and provide transformative learning experiences for students (Derosa & Jhangiani, 2017; Lubicz‐Nawrocka & Bovill, 2023; Miglietti & Strange, 1998). For example, students weighed assessments differently, with discussion board posts being the lowest. Students may have chosen lower percentages because they were nervous about the score they would receive, did not prioritize work or growth in an area, or were not planning to spend much time on that category. Students also distributed weight differently than the instructor, with a notably lower weight in coding exercises. Students demonstrated relief at the opportunity to weight their assessments, which seemed to reduce their perceived threat of failure (Bledsoe & Baskin, 2014). Regardless of the reason behind the grade weighting, this provided agency and allowed students to prioritize their own learning goals. Kaw (2020) found that 16% of students in their numerical methods course received a higher final grade with their weighting criteria than the instructor's. Students overwhelmingly (e.g., 4.7/5, Lang, 2018) appreciate the opportunity to choose the weighting of assessments.

Open science and open education synergisms can excite students about science processes. Students in this course reported gains in their confidence in comprehension, comfort with complex ideas, and enthusiasm for data analyses. These reported gains are essential to consider in contributing to the students' learning outcomes because studies found that their comfort level was the best predictor of student success in learning programming (Wilson & Shrock, 2001). Truan and Dressel (2022) found that undergraduate students favored the ideals and values of open science, and repeated exposure to open education practices in curricula increases students' willingness to engage in future open science practices. Furthermore, including student experiences and sentiments around open science and education practices provides additional inclusion of diverse voices and perspectives, thereby promoting inclusion in open science and fostering students' scientific identity (Grahe et al., 2020).

### 5.1 Recommendations

Open science can still be improved to broaden its use and applicability in undergraduate education. If the goal of open science is to share knowledge with the public (Eppinger, 2021), then more consideration for teaching scientists could be supported (Schaffert & Geser, 2008). In this study, I found that datasets adhered to about half of the fairness practices I assessed, but this adherence improved over time. Practices least used by authors included providing code for statistical analyses, providing enough information for an undergraduate student to understand the steps, and including qualified references to other (meta)data. Also, I recognize that not every publication and dataset considers the application of their information and data in an educational context, and we appreciate and commend all authors for publicly opening their articles and corresponding datasets. I encourage authors, publications, and granting agencies to consider and expand the fairness guides and practices they apply to their articles and datasets (Filazzola & Lortie, 2022; Jenkins et al., 2023; Wilkinson et al., 2016) while considering undergraduate and graduate students as potential audiences.

In this course, I used open science components (i.e., openly licensed literature and datasets) as an OER in a larger open education framework. In turn, the work from this course (this manuscript and datasets) may be used by others as a teaching and research resource, as they exemplify open learning principles of “collaboration and sharing of information, connected communication about learning and teaching, collectivity to grow knowledge and resources, critique for the promotion of scholarship and serendipitous innovation” (Conole, 2013 as cited in Nascimbeni, 2020, p. 125). Continued examination of the use and effectiveness of open education by instructors using and developing these resources can simultaneously contribute to the three areas of open education: open science, open innovation, and open research (Ramirez‐Montoya, 2020).

In general, because undergraduate students are learning and practicing multiple aspects of science practices, adding greater transparency and clarity in all steps and components would be helpful. In particular, four key recommendations arose from student reflections. First, include explicit connections between goals, hypotheses, and analyses, including key terms students can use to recognize these components (e.g., dependent and independent variables). Next, excluding sensitive data, include all data in the dataset used in article analyses so students can practice recreating different analyses. This is particularly important for less complex statistical analyses that may be taught at the undergraduate level, like those found in McDonald (2014). Third, add more details to the (meta)data. For example, scientists often use abbreviations for their data variable headers in a spreadsheet but then include little to no explanation of what the abbreviation stands for or how that variable was measured. Finally, undergraduate students are most fearful and least practiced in writing code to conduct statistical analyses, so including a supplemental file or tab in a spreadsheet with code may facilitate them learning these components.

### 5.2 Limitations and additional considerations

This study highlights examples and experiences from a small group of students in a capstone course at a STEM institution. This small sample size did not allow me to consider how the intersections of identities (e.g., gender, race, ethnicity, and more) may influence their perceived learning gains or reflective practices. In addition, all students were of similar majors (i.e., Biology), so extrapolating these findings to all STEM disciplines is beyond the scope of this work. In addition, students at a STEM institution may exhibit lower levels of math anxiety than their peers in non‐STEM programs or institutions (Daker et al., 2023). This positive familiarity with math may have influenced their learning gains, like their attitudes or enthusiasm for data analyses. However, note that many students shared that although they may have taken a statistics or coding course before this capstone, most of them felt inadequately prepared for and nervous at the start of the course.

Furthermore, the small class size allowed for a flexible course design and instruction, which allowed for a high level of interaction between instructors and students, time for the instructor to provide rapid and personalized feedback, and accommodate different learning styles of students. Small class sizes, especially when supporting marginalized populations (e.g., women in computer science), have been documented to be perceived as being more welcoming, inclusive, and supportive (Ying et al., 2021). Typically, time, interaction, and flexibility constraints increase with course size, increasing students' fears and frustrations and decreasing their learning gains, especially when considering the complexity of learning and executing statistical analyses and coding (Jenkins, 2002; Rogerson & Scott, 2010). In addition, early career and minoritized faculty often have heavy teaching loads or large class sizes that reduce their ability to implement similar structures or practices in their courses (Grahe et al., 2020; Kathawalla et al., 2021; Pownall et al., 2021; Reidinger, 2022).

To facilitate this exploratory study of themes and sentiments of students in their reflections about open science and education practices, I used an autocoding feature in thematic analyses. Because autocoding works at the level of words, sentences or phrases with multiple words were found across multiple themes or sentiments. The same sentence could be referenced multiple times in the same or different theme or sentiment, which made interpretation of the themes and their sentiments challenging due to the high number of references. In addition, words may have been miscoded in some cases – as was when a student wrote about “human–wildlife conflict” as a topic, but the autocoding included conflict as a negative sentiment. These issues demonstrate the complexity of the information in the reflections, the limitations of autocoding, and the need for the human analyst to sift through and add interpretation to the results.

### 5.3 Conclusion

Finding primary literature and corresponding datasets that meet a course's learning objectives can be challenging (Rauschert et al., 2011). In the current state of the open science movement, where vast amounts of data are being published in connection to journal articles, these resources can be valuable for educators (Schaffert & Geser, 2008). However, more research on the use, utility, and effectiveness of open science, in the form of primary literature and data, in meeting student learning outcomes, is needed to provide additional recommendations and educational benefits to the open science community, particularly considering that multiple issues compounded the frustrations of students. Publishing on open science and open education practices is a great way to recognize effective teaching and learning and advance the careers of students and faculty, especially early career and teaching faculty (Power, 2021). Fortunately, there are numerous ways to integrate open science with open education, and benefits can reach beyond the classroom by boosting accessibility and reproducibility for all scientists (McKiernan et al., 2016; Tennant et al., 2016).

## AUTHOR CONTRIBUTIONS

**Marja H. Bakermans:** Conceptualization (lead); data curation (lead); formal analysis (lead); investigation (lead); methodology (lead); visualization (lead); writing – original draft (lead); writing – review and editing (lead).

## CONFLICT OF INTEREST STATEMENT

I have no competing or conflicts of interest to disclose.

## Data Availability

The data supporting this study's findings are openly available in the Dryad data repository at https://doi.org/10.5061/dryad.37pvmcvst (private for peer review link: https://datadryad.org/stash/share/mBXcaXv51YpQXBXYKMN2gZq1PGhY0TWGxx1U0iIzkqs). In respect of student privacy, entire reflection essays were not included in the public repository and only excerpts from student essays were included in this paper.

## APPENDIX A THE BASIC STRUCTURE OF THE CAPSTONE COURSE WITH THE TOPICS (E.G., WHAT TYPES OF ANALYSES WILL BE COVERED) AND THE COURSE WORK TO BE COMPLETED (E.G., READINGS, EXERCISES, ETC.). THIS SCHEDULE IS POSTED ON THE HOME LANDING PAGE OF THE LMS COURSE PAGE

**Table:**

Session	Topic	Coursework
1	Course Introduction Introduction to R, Dryad, article, and dataset	Readings 1: Introduction to R and Foundational and Applied Statistics Using R Grading Activity/Discussion Board
2	Basics in data analysis Dataset: empty forests	Readings 2: Chapters on Basics (analysis steps) Article: Empty forests Exercise 1
3 and 4	Descriptive statistics & Graphing Dataset: barn owl data	Readings 3: Chapters on Basics cont. (hypotheses) Readings 4: Chapters on Descriptive statistics Article: Barn owl habitat selection Exercise 2
5 and 6	Tests for nominal variables Dataset: crop‐raiding data	Readings 5: Chapters on tests for nominal variables Paper: Crop raiding by large mammals Exercise 3 Team 1 Lead Assignment
7 and 8	Tests for one measurement variable Dataset: foraging birds data	Readings 7: Chapters on tests for one measurement variable Article: Foraging ecology of shrubland birds Exercise 4 Team 2 Lead Assignment
9 and 10	Measurement variables, part 2 Dataset: koala foraging data	Readings 9: Chapters on tests for multiple measurement variables Article: Foraging patterns of koalas Exercise 5 Team 3 Lead
11 and 12	Nonparametric tests Dataset: arthropod dataset	Reading 11 s: Chapters on nonparametric tests Article: Arthropods of the great indoors Exercise 6 Team 4 Lead
13 and 14		Final project due Statement of Learning reflection Learning gains survey

## APPENDIX B JOURNAL ARTICLES, THEIR CORRESPONDING DATASETS, AND HOW EACH WAS USED IN THE CAPSTONE COURSE, *ANALYSES IN WILDLIFE ECOLOGY AND CONSERVATION*

**Table:**

Journal article	Corresponding dataset	Use in class
Bogoni, J. A., et al. (2023). The empty forest three decades later: Lessons and prospects. *Biotropica*, 55, 13–18. https://doi.org/10.1111/btp.13188	Bogoni, J. A., et al. (2022). Dataset for: The empty forest three decades later: Lessons and prospects [Dataset]. *Dryad*. https://doi.org/10.5061/dryad.8kprr4xs4	Exercise 1 – load data into R, simple plot
Castañeda, X. A., et al. (2021). Barn Owls select uncultivated habitats for hunting in a winegrape growing region of California, *Ornithological Applications*, 123(1), duaa058. https://doi.org/10.1093/ornithapp/duaa058	Johnson, M. (2021). Data from: Barn Owls select uncultivated habitats for hunting in a winegrape growing region of California [Dataset]. *Dryad*. https://doi.org/10.5061/dryad.mpg4f4qx0	Exercise 2 – descriptive statistics
Mamo, A., et al. (2021). Pattern of crop raiding by wild large mammals and the resultant impacts vary with distances from forests in Southwest Ethiopia. *Ecology and Evolution*, 11: 3203–3209. https://doi.org/10.1002/ece3.7268	Lemessa, D. (2021). Pattern of crop raiding by wild large mammals and the resultant impacts vary with distances from forests in southwest Ethiopia [Dataset]. *Dryad*. https://doi.org/10.5061/dryad.tht76hdz3	Exercise 3 – tests for nominal variable (e.g., chi‐square test of goodness of fit)
Guernsey, N. C., et al. (2023). Post‐translocation dynamics of black‐tailed prairie dogs (*Cynomys ludovicianus*): A successful conservation and human–wildlife conflict mitigation tool. *Ecology and Evolution*, 13, e9738. https://doi.org/10.1002/ece3.9738	Guernsey, N. C., et al. (2023). Post‐translocation dynamics of black‐tailed prairie dogs (*Cynomys ludovicianus*): A successful conservation and human‐wildlife conflict mitigation tool [Dataset]. *Dryad*. https://doi.org/10.5061/dryad.9zw3r22j9	Team Lead 1
Deshwal, A., et al. (2022). Foraging habitat selection of shrubland bird community in tropical dry forest. *Ecology and Evolution*, 12(8). https://doi.org/10.1002/ece3.9192	Deshwal, A. (2022). Dataset on foraging ecology of shrubland bird community [Dataset]. *Dryad*. https://doi.org/10.5061/dryad.7m0cfxpxb	Exercise 4 – tests for one measurement variable (normality and transformation)
Munguia, P., et al. (2017). Thermal constraints on microhabitat selection and mating opportunities. *Animal Behavior*, 123, 259–265. https://doi.org/10.1016/j.anbehav.2016.11.004	Munguia, P., et al. (2017). Data from: Thermal constraints on microhabitat selection and mating opportunities [Dataset]. *Dryad*. https://doi.org/10.5061/dryad.ph416	Team Lead 2
Crowther, M. S., et al. (2022). Patch quality and habitat fragmentation shape the foraging patterns of a specialist folivore. *Behavioral Ecology*, 33(5), 1007–1017. https://doi.org/10.1093/beheco/arac068	Crowther, M., et al. (2022). Patch quality and habitat fragmentation shape the foraging patterns of a specialist folivore [Dataset]. *Dryad*. https://doi.org/10.5061/dryad.0p2ngf249	Exercise 5 – measurement variables, cont. (e.g., linear regression)
Jha, R. R. & Isvaran, K. (2022). Antelope space‐use and behavior indicate multilevel responses to varying anthropogenic influences in a highly human‐dominated landscape. *Ecology and Evolution*, 12(10), e9372–n/a. https://doi.org/10.1002/ece3.9372	Jha, R. R. & Isvaran, K. (2022). Antelope space‐use and behavior indicate multi‐level responses to varying anthropogenic influences in a highly human‐dominated landscape [Dataset]. *Dryad*. https://doi.org/10.5061/dryad.jq2bvq89k	Team Lead 3
Bertone M. A., et al. (2016). Arthropods of the great indoors: characterizing diversity inside urban and suburban homes. *PeerJ*, 4:e1582. https://doi.org/10.7717/peerj.1582	Raw Data File is embedded within the article: https://doi.org/10.7717/peerj.1582/supp‐2	Exercise 6 – nonparametric tests (e.g., Kruskal–Wallis test)
Martin, M., et al. (2022). Irregular forest structures originating after fire: An opportunity to promote alternatives to even‐aged management in boreal forests. *The Journal of Applied Ecology*, 59(7), 1792–1803. https://doi.org/10.1111/1365‐2664.14186	Martin, M. (2022). Data from: Irregular forest structures originating after fire: an opportunity to promote alternatives to even‐aged management in boreal forests [Dataset]. Dryad. https://doi.org/10.5061/dryad.2v6wwpzqx	Team Lead 4
Ikemoto, M., et al. (2023). Diurnal and geographic variations of pollinator importance for Cucurbita maxima Duchesne. *Ecology and Evolution*, 13(11), e10651–e10651. https://doi.org/10.1002/ece3.10651	Ikemoto, M., Tanaka, Y., Kohno, K., Yokoi, T. (2023). Diurnal and geographic variations of pollinator importance for Cucurbita maxima Duchesne [Dataset]. *Dryad*. https://doi.org/10.5061/dryad.vmcvdnczm	Final Project
Gulick, A. G., et al. (2022). An underwater Serengeti: Seagrass‐mediated effects on intake and cultivation grazing behavior of a marine megaherbivore. *Ecosphere*, *13*(11). https://doi.org/10.1002/ecs2.4259	Gulick, A. G., et al. (2022). An underwater Serengeti: Seagrass‐mediated effects on intake and cultivation grazing behavior of a marine megaherbivore [Dataset]. *Dryad*. https://doi.org/10.5061/dryad.g4f4qrfsx	Final Project
Everaert, G., et al. (2014). Comparison of the abiotic preferences of macroinvertebrates in tropical river basins*. PLoS ONE*, 9(10), e108898. https://doi.org/10.1371/journal.pone.0108898	Everaert, G., et al. (2015). Data from: Comparison of the abiotic preferences of macroinvertebrates in tropical river basins [Dataset]. *Dryad*. https://doi.org/10.5061/dryad.20860	Final project
Hinderer, R. K., et al. (2021). Habitat selection by a threatened desert amphibian. *Ecology and Evolution*, 11(1), 536–546. https://doi.org/10.1002/ece3.7074	Hinderer, R. (2021). Habitat Selection by a Threatened Desert Amphibian [Dataset]. *Dryad*. https://doi.org/10.5061/dryad.h9w0vt4gp	Final project
Fragoso, F. P., et al. (2021). Patch selection by bumble bees navigating discontinuous landscapes. *Scientific Reports*, 11(1), 8986–8986. https://doi.org/10.1038/s41598‐021‐88394‐2	Brunet, J., Fragoso, F. (2023). Data for: Patch fidelity and patch preference of honey bees and bumble bees [Dataset]. *Dryad*. https://doi.org/10.5061/dryad.zcrjdfnj4	Final project
Hinderer, R. K., et al. (2020). Habitat selection by a threatened desert amphibian. *Ecology and Evolution*, 11(1), 536–546. https://doi.org/10.1002/ece3.7074	Hinderer, R. (2021). Habitat Selection by a threatened desert amphibian [Dataset]. *Dryad*. https://doi.org/10.5061/dryad.h9w0vt4gp	Final project (used by 2 students)
Koziol, L., et al. (2022). Native mycorrhizal fungi improve milkweed growth, latex, and establishment while some commercial fungi may inhibit them. *Ecosphere (Washington, D.C)*, 13(5). https://doi.org/10.1002/ecs2.4052	Koziol, E. (2022). Data for Native mycorrhizal fungi improve milkweed growth, latex, and establishment while some commercial fungi may inhibit them [Dataset]. *Dryad*. https://doi.org/10.5061/dryad.hdr7sqvk4	Final project
Matthews, L. P., et al. (2020). Acoustically advertising male harbor seals in southeast Alaska do not make biologically relevant acoustic adjustments in the presence of vessel noise. *Biology Letters*, 16(4), 20,190‐795. https://doi.org/10.1098/rsbl.2019.0795	Matthews, L. P., et al. (2020). Data from: Acoustically advertising male harbor seals in southeast Alaska do not make biologically relevant acoustic adjustments in the presence of vessel noise [Dataset]. *Dryad*. https://doi.org/10.5061/dryad.pvmcvdnh8	Final project
Baumberger, K. L., et al. (2019). Movement and habitat selection of the western spadefoot (*Spea hammondii*) in southern California. *PLoS ONE*, 14(10), e0222532. https://doi.org/10.1371/journal.pone.0222532	Baumberger, K., et al. (2020). Data from: Movement and habitat selection of the western spadefoot (*Spea hammondii*) in southern California [Dataset]. *Dryad*. https://doi.org/10.5061/dryad.8359820	Final project
Cruz, J., et al. (2018). Managing individual nests promotes population recovery of a top predator. *The Journal of Applied Ecology*, 55(3), 1418–1429. https://doi.org/10.1111/1365‐2664.13062	Cruz, J., et al. (2018). Data from: Managing individual nests promotes population recovery of a top predator [Dataset]. *Dryad*. https://doi.org/10.5061/dryad.8ff85	Final project
Tremlett, C. J., et al. (2020). Pollination by bats enhances both quality and yield of a major cash crop in Mexico. *The Journal of Applied Ecology*, 57(3), 450–459. https://doi.org/10.1111/1365‐2664.13545	Tremlett, C., et al. (2019). Pollination by bats enhances both quality and yield of a major cash crop in Mexico [Dataset]. *Dryad*. https://doi.org/10.5061/dryad.dr7sqv9v2	Final project
Montiglio, P. ‐O., & DiRienzo, N. (2016). There's no place like home: the contribution of direct and extended phenotypes on the expression of spider aggressiveness. *Behavioral Ecology*, arw094. https://doi.org/10.1093/beheco/arw094	Montiglio, P. ‐O., & DiRienzo, N. (2016). Data from: There's no place like home: the contribution of direct and extended phenotypes on the expression of spider aggressiveness [Dataset]. *Dryad*. https://doi.org/10.5061/dryad.ng4b0	Final paper
Dinh, Q. M., et al. (2022). Reproduction ecology of an emerging fishery resource, the amphibious mudskipper *Periophthalmus chrysospilos*, in the Mekong Delta. *Ecology and Evolution*, *12*(1), e8507–n/a. https://doi.org/10.1002/ece3.8507	Dinh, Q. (2023). Reproduction ecology of an emerging fishery resource, the amphibious mudskipper *Periophthalmus chrysospilos*, in the Mekong Delta [Dataset]. *Dryad*. https://doi.org/10.5061/dryad.2fqz612q8	Final paper
